# Supplementary material for: Self-Assembly of Au-Fe3O4 Hybrid Nanoparticles Using a Sol–Gel Pechini Method
Source: Molecules. 2021 Nov 17;26(22):6943. doi: 10.3390/molecules26226943 (PMC8624103; doi:10.3390/molecules26226943)
Supplement: Supplementary file 1 [file molecules-26-06943-s001.zip › molecules-1440879-supplementary.pdf]

Supporting Information: Self-assembly of Au-Fe_3O_4 hybrid nanoparticles using a sol-gel Pechini method

Jesus G. Ovejero[1,2,*], Miguel A. García[1,2] and Pilar Herrasti[3]

[1] Instituto de Magnetismo Aplicado, 'Salvador Velayos', UCM-CSIC-ADIF, Las Rozas, PO Box 155, Madrid 28230, Spain.

[2] Department of Dosimetry and Radioprotection, Hospital General Universitario Gregorio Marañón, 28007 Madrid, Spain.

[3]Instituto de Cerámica y Vidrio, ICV-CSIC, C/ Kelsen 5, Cantoblanco, 28049, Madrid, Spain.

[4]Departamento de Química Física Aplicada, Facultad de Ciencias, Universidad Autónoma de Madrid, Cantoblanco s/n, 28049 Madrid, Spain

**Corresponding author: jgovejero@ucm.es;*

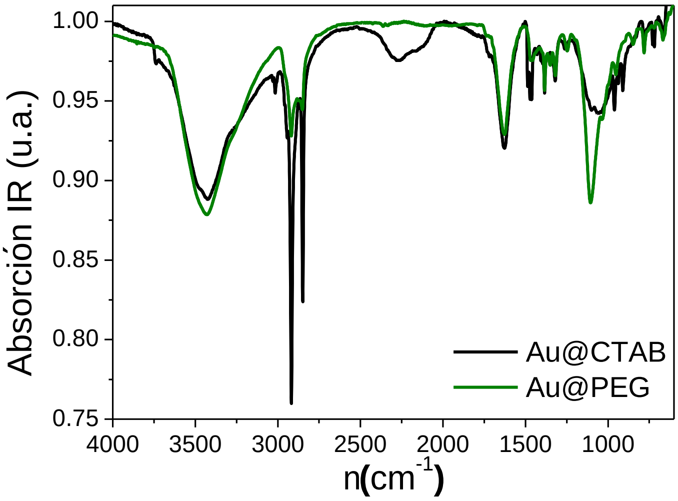

Figure S1. *FT-IR transmission spectrum of CTAB coated AuNR (Au@CTAB) in black and PEG coated AuNR (Au@PEG) in green.*

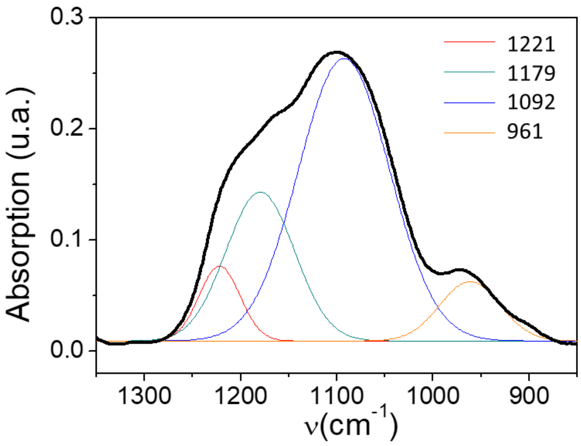

Figure S2. *Deconvolution of 1105 cm^{-1} peak of the FT-IR spectrum in hybrid nanostructures.*

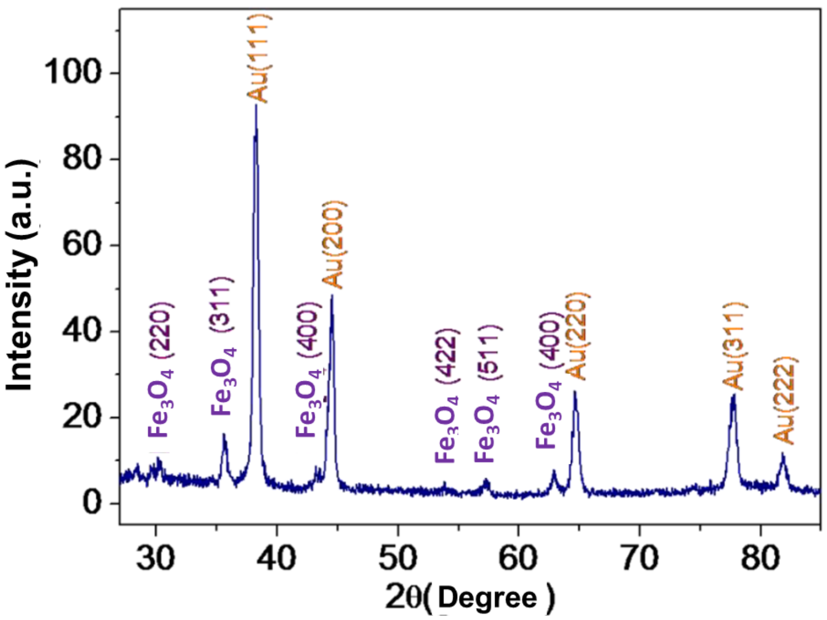

Figure S3. *XRD pattern of hybrid nanostructures. Fe_3O_4 and Au characteristic peaks appear labeled in purple and yellow, respectively.*
